# Raman‐activated sorting of antibiotic‐resistant bacteria in human gut microbiota

**DOI:** 10.1111/1462-2920.14962

**Published:** 2020-03-13

**Authors:** Yi Wang, Jiabao Xu, Lingchao Kong, Bei Li, Hang Li, Wei E. Huang, Chunmiao Zheng

**Affiliations:** ^1^ School of Environment Harbin Institute of Technology Harbin 150090 China; ^2^ Guangdong Provincial Key Laboratory of Soil and Groundwater Pollution Control, School of Environmental Science and Engineering Southern University of Science and Technology Shenzhen 518055 China; ^3^ Department of Engineering Science University of Oxford, Parks Road Oxford OX1 3PJ UK; ^4^ The State Key Lab of Applied Optics Changchun Institute of Optics, Fine Mechanics and Physics, CAS 130033 Changchun China; ^5^ HOOKE Instruments Ltd. 130033 Changchun China

## Abstract

The antibiotic‐resistant bacteria (ARB) and antibiotic‐resistant genes (ARGs) in human gut microbiota have significant impact on human health. While high throughput metagenomic sequencing reveals genotypes of microbial communities, the functionality, phenotype and heterogeneity of human gut microbiota are still elusive. In this study, we applied Raman microscopy and deuterium isotope probing (Raman–DIP) to detect metabolic active ARB (MA‐ARB) *in situ* at the single‐cell level in human gut microbiota from two healthy adults. We analysed the relative abundances of MA‐ARB under different concentrations of amoxicillin, cephalexin, tetracycline, florfenicol and vancomycin. To establish the link between phenotypes and genotypes of the MA‐ARB, Raman‐activated cell sorting (RACS) was used to sort MA‐ARB from human gut microbiota, and mini‐metagenomic DNA of the sorted bacteria was amplified, sequenced and analysed. The sorted MA‐ARB and their associated ARGs were identified. Our results suggest a strong relation between ARB in human gut microbiota and personal medical history. This study demonstrates that the toolkit of Raman–DIP, RACS and DNA sequencing can be useful to unravel both phenotypes and genotypes of ARB in human gut microbiota at the single‐cell level.

## Introduction

The human intestine accommodates a large number of intestinal bacteria (Hao and Lee, 2004) which exert significant physiological functions including assisting host digestion and immunity, synthesis of essential vitamins such as vitamin K and resistance to pathogen invasion (O'Hara and Shanahan, 2006; Blaser, 2011; Iliev *et al*., 2012; Mills *et al*., 2013; Hillman *et al*., 2017; Thursby and Juge, 2017). The effects of antibiotics on the intestinal microbiota have been extensively studied for several decades following the emergence of antibiotic resistance within the indigenous bacterial communities in the gut (Modi *et al*., 2014). It has been reported that the use of antibiotics has a great and long‐lasting influence on the diversity, structure and function of intestinal microbiota (Dethlefsen and Relman, 2011; Reijnders *et al*., 2016), which can lead to prevalence of ARB, ARG and risks of many diseases such as obesity, diabetes, and inflammatory bowel disease (Blaser, 2011; Almagor *et al*., 2018). ARB has a significant impact on human health and a recent medical case reported that a transmitted antibiotic‐resistant *Escherichia coli* (*E*. *coli*) killed a patient after treated with faecal microbiota transplant (DeFilipp *et al*., 2019). Therefore, the analysis and identification of ARB in human gut microbiota are important to clinic practice and precision medicine.

Conventional cultivation method is powerful to isolate and study antibiotic‐resistant bacteria from human gut microbiota, but only 20%–40% of the microorganisms can be cultured *in vitro* (Amann *et al*., 1995; Thursby and Juge, 2017). Even though these bacteria can be cultivated, it would be important to put the bacteria in their biological context, and study the metabolism and function in their natural habitats. This emphasized the need for culture‐independent approach to study bacteria in complex environments such as human gut. The advances in omics techniques, especially high‐throughput metagenomic sequencing, has been accelerating studies of the human intestinal microbiome, including exploring phylogenetic diversity and identifying functional genes (Wang *et al*., 2015). However, bulk omics technologies like genomics, transcriptomics and proteomics could miss important metabolic activity, functionality and phenotypic heterogeneity of bacteria (Hu *et al*., 2016; Goldman *et al*., 2019). Human microbiota usually contains both ARB and susceptible bacteria, it is informative to investigate how individual members response to a drug treatment, especially when ARB is a minority group within the population. On top of that, the phenotype–genotype association of functional ARB is still poorly characterized. It is still very difficult to investigate the role of bacteria in a complex community such as the gut microbiota and to link functions and phenotypes to genotypes.

Single‐cell Raman micro‐spectroscopy is a non‐invasive profiling technology to reveal metabolic activity of single cells without externally tagging molecules (Huang *et al*., 2009; Zhang *et al*., 2015a; McIlvenna *et al*., 2016; Song *et al*., 2016; Song *et al*., 2017b; Jing *et al*., 2018; Lee *et al*., 2019). Raman microscopy with deuterium isotope probing (Raman–DIP) has been demonstrated to *in situ* identify the metabolic activity and pathway of single cells of bacterial cultures, intestinal bacteria in mouse caecum, bacteria in human faeces, and ARB in river water (Henk‐Jan *et al*., 2008; Berry *et al*., 2015; Wang *et al*., 2016; Xu *et al*., 2017; Wang *et al*., 2019). Heavy water or D_2_O is an effective DIP probe for Raman detection of ARB, as metabolically active cells can incorporate deuterium (D) from D_2_O into their biomass via NADPH mediated H/D exchange reactions and produce a distinct C–D band at the Raman region of 2000–2300 cm^−1^, shifted from the C–H band at 2800–3200 cm^−1^ (Berry *et al*., 2015). When the concentration of antibiotics is high enough, the metabolic activity of the susceptible bacteria will be inhibited, but not to those of ARB, which will actively generate C–D band in the single‐cell Raman spectra (SCRS) of ARB (Berry *et al*., 2015; Song *et al*., 2017a). RACS is an emerging cell‐sorting approach, using SCRS as the sorting criteria. A significant advantage of the toolkit (Raman–DIP and RACS) is to *in situ* detection, identify and sort the active bacteria at a single‐cell level in a complex environment (Berry *et al*., 2015; Cui *et al*., 2018).

In this study, we employed Raman–DIP, Raman‐activated cell ejection (RACE, one of the methods of RACS) and metagenomic sequencing to study the relative abundance of ARB, and the functional ARG of the sorted bacteria in human gut microbiota from two volunteers. Our study suggests that the abundance of ARB in gut is related to personal exposure to antibiotics by administering medicine. By combining Raman sorting of the ARB and genomic sequencing, we establish a link between bacterial phenotypes and genotypes that translates our understanding in the complex environment of human intestinal microbiota.

## Results and discussion

### *In situ* identification of MA‐ARB in human microbiota by Raman–DIP

**Figure 1:**
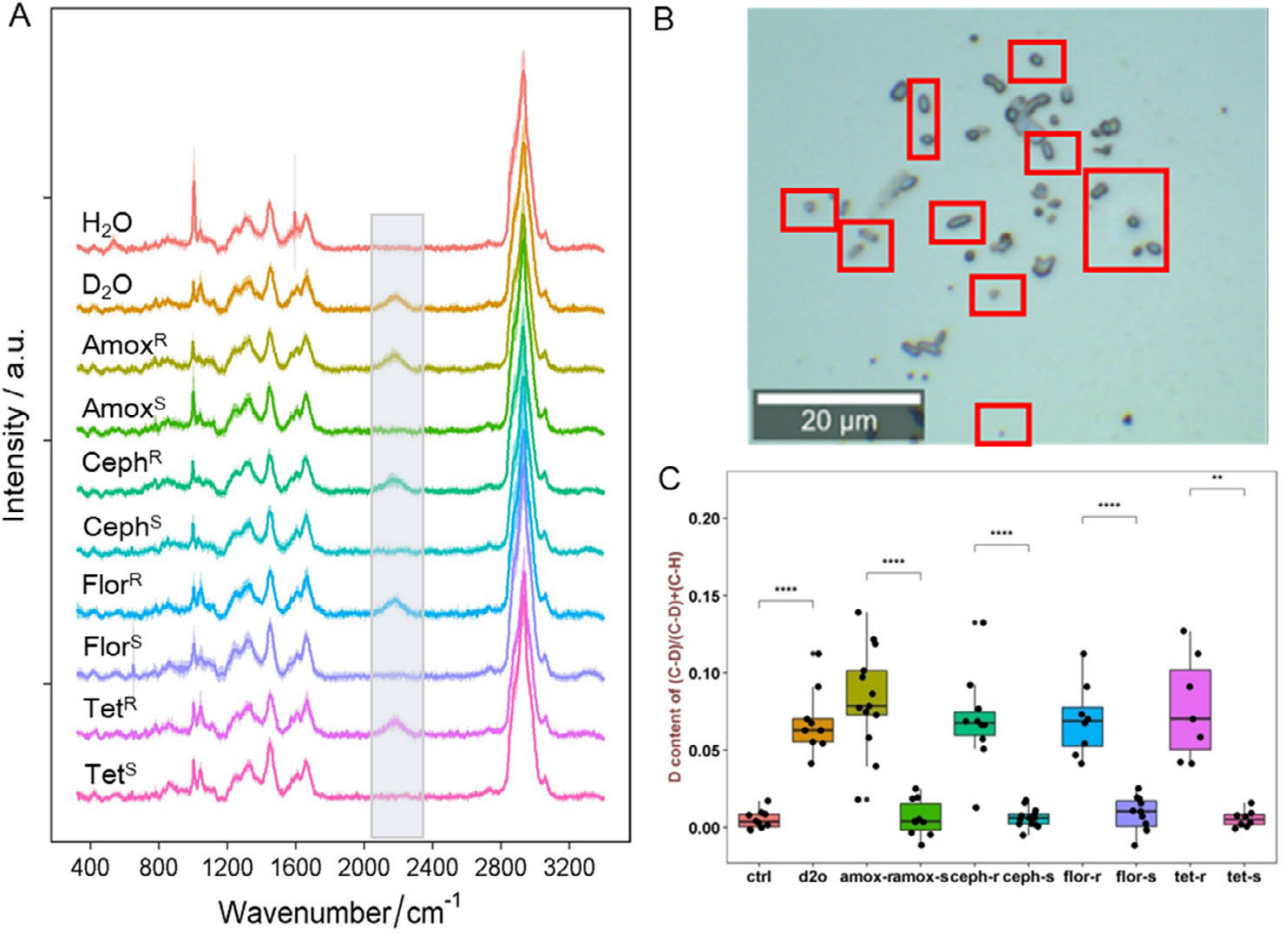
A. Single‐cell Raman spectra (SCRS) of human intestinal bacteria after *in situ* culturing with H_2_O, D_2_O, D_2_O + amoxicillin, D_2_O + cephalexin, D_2_O + florfenicol or D_2_O + tetracycline for 24 h at 37°C anaerobically (R: metabolic active resistant strain; S: susceptible strain). The C–D band (2070–2300 cm^−1^) is highlighted. Each spectrum represents an average of SCRS from 80 to 300 single‐cells, and the shadow represents standard deviation of SCRS.B. A microscopic field view of intestinal bacteria community with D_2_O + cephalexin (2 × MIC), showing the microorganisms with C–D metabolic activity (red squares).C. The intensity ratio of C–D/(C–D + C–H) in each SCRS of bacteria cells in Fig. 1A. Student *t*‐test was used to indicate statistical differences between R and S strain. *P < 0.05, **P < 0.01, ***P < 0.001, ****P < 0.0001.

Figure 1A shows the average SCRS of 80–300 single human intestinal bacteria with standard deviation. These bacteria in faeces were cultivated for 24 h at 37°C anaerobically with H_2_O, D_2_O, D_2_O with amoxicillin, D_2_O with cephalexin, D_2_O with florfenicol, and D_2_O with tetracycline. Usually, SCRS of bacteria (e.g. in the case of bacteria incubated with H_2_O) show no signal in the range of 1800–2700 cm^−1^, so‐called ‘silent zone’. The metabolically active bacteria incubated with D_2_O display a clearly distinguishable band at 2040–2300 cm^−1^ (Fig. 1A), due to the formation of C–D chemical bonds via the exchange of H^+^ to D^+^ in NADPH/NADH^+^ during cell metabolism (Berry *et al*., 2015). Therefore, the C–D band can be used as an indicator of single cell activity. Most of the bacteria (63%–80%) in the faeces with D_2_O showed a C–D band in their SCRS, indicating active metabolism of intestinal bacteria (Fig. 1A).

By cultivating faecal samples with D_2_O in the presence of different antibiotics, the ‘resistant’ and ‘susceptible’ bacteria in human gut microbiota can be distinct by displaying the presence (metabolically active) and absence (metabolically inactive) of a C–D band in their SCRS (Fig. 1A). It is expected that the addition of antibiotics greater than minimal inhibitory concentration (MIC) could have a strong inhibitory effect on the growth and biological activity of the susceptible bacteria. Five antibiotics were used in this study. Tetracycline and florfenicol affect the elongation of peptide chains and protein synthesis thus inhibiting bacterial growth (Chukwudi, 2016). Amoxicillin, cephalexin and vancomycin inhibit cell wall synthesis, leading to the expansion of the cell content and cell disruption, consequently causing cell death (Cho *et al*., 2014). Antibiotic inhibitive and killing effects can stop or disrupt bacterial metabolism, preventing from the formation of C–D chemical band. Therefore, C–D Raman band can be used as a biomarker to identify the MA‐ARB in human gut microbiota by incubating the bacterial community with D_2_O and antibiotics at the same time (Xu *et al*., 2017).

According to C–D band in SCRS (Fig. 1A), ARB were identified and counted in the samples from two human gut microbiota. Figure 1B shows an example of the microscopic field view of the human gut bacteria under the treatment of cephalexin (2 × MIC), which have different shapes and sizes. Ceph^R^ and Ceph^S^ bacteria could not be distinguished by their morphology under the microscope, as intestinal bacteria are highly diverse and have different sizes and shapes. However, bacteria with active metabolism could be detected by identifying the presence of a C–D band in their SCRS (red squares in Fig. 1B).

The ratio of C–D/(C–D + C–H) represents the incorporation of deuterium (D), which can be used as a semi‐quantitative indicator of bacterial general metabolic activity (Berry *et al*., 2015; Xu *et al*., 2017). As shown in Fig. 1C, the C–D ratio in SCRS of MA‐ARB was significantly higher than the susceptible bacteria (*P* < 0.0001), for all antibiotics. Interestingly, the C–D ratio of MA‐ARB treated with D_2_O and antibiotic kept at a similar level compared with the bacteria only treated with D_2_O (Fig. 1C), suggesting that the addition of antibiotics to those MA‐ARB did not have any significant impact on their metabolic activities. ARB can resist antibiotics by horizontal gene transfer (e.g. vancomycin), rRNA target alteration (e.g. florfenicol), decrease of antibiotic uptake via efflux pump of antibiotics (e.g. amoxicillin, cephalexin tetracycline) and alternation of protein synthesis and/or induction of the modifying enzyme (Schnappinger and Hillen, 1996; Gardete and Tomasz, 2014; Chukwudi, 2016). Since the resistant mechanism is energetic process, it might contribute to the intensity of the C–D band.

While some studies demonstrated that the fingerprint region of Raman spectra (200–1800 cm^−1^) were able to analyse the bacterial phenotype under antibiotic influences, and determine the MIC and resistant mechanisms of *E*. *coli* in different antibiotics (Athamneh *et al*., 2014; Teng *et al*., 2016; Tao *et al*., 2017; Xu *et al*., 2017; Germond *et al*., 2018; Kirchhoff *et al*., 2018; Novelli‐Rousseau *et al*., 2018), the C–D band from SCRS is a simple and distinguishable Raman biomarker to identify the MA‐ARB in environmental samples (Xu *et al*., 2017). In this study, we demonstrated that the C–D band can be used to identify the MA‐ARB in human gut microbiota. This approach can effectively and rapidly identify the bacterial metabolic activity and detect the functional MA‐ARB not only in culture medium, but also in real and complex human microbiota at a single‐cell level.

### Detection of abundance of MA‐ARB in human intestine related to personal lifestyles

**Figure 2:**
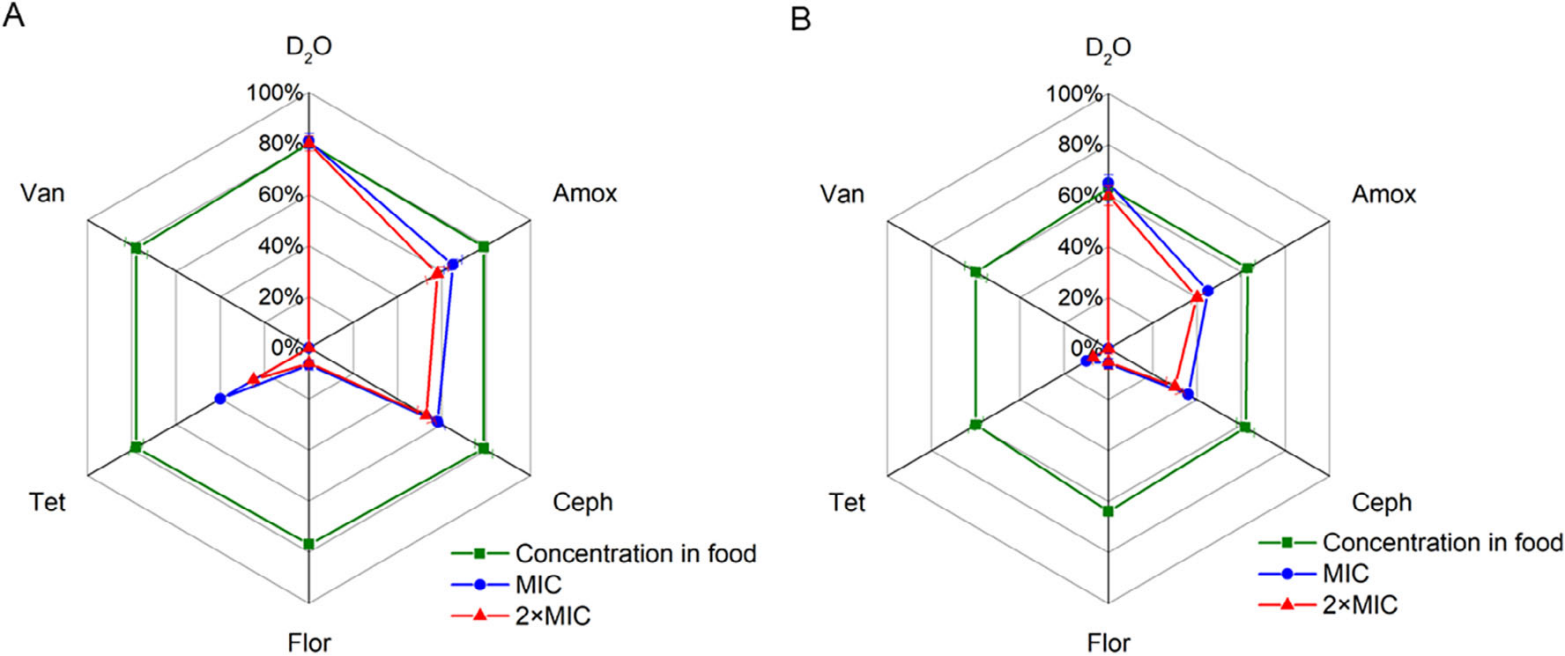
A and B. Percentages of active intestinal microbiota with a response of different antibiotics in two volunteers, cultured *in situ* with the concentration of antibiotics at food residual level, MIC or 2 × MIC. This percentage is regarded as the abundance of MA‐ARB in the community. For each condition, three samples were measured with 80–300 cells per sample. The error bar represents standard deviation from three samples.

Figure 2A and B show the active ratios of bacteria in the gut microbiota from the two volunteers. The faecal samples were treated with five different antibiotics (Table 1). SCRS of bacteria in human gut microbiota, and the intensities of the C–D band under different antibiotic treatments, are shown in Supporting Information Fig. S1. The abundances of active bacteria were calculated by counting the number of bacteria displaying C–D band among the total measured SCRS in samples (Xu *et al*., 2017). The D_2_O condition without adding antibiotics represented the average active percentage, revealing that 80% and 63% of the bacteria were active in the microbial communities of the two volunteers. The difference is conceivable as different diet and living environment between individuals (Conlon and Bird, 2015). Exposure to a range of antibiotics caused a great change on the active ratio of gut microbiota. The abundance of MA‐ARB resistant to different antibiotics was significantly different (Fig. 2A and B). Little difference in the active ratios was observed between samples treated with MIC and those with 2 × MIC concentrations of antibiotics. This is similar to the previous study showing that there was no significant difference between the bacteria in the river treated with MIC and 10 × MIC concentrations of antibiotics (Xu *et al*., 2017). In the following discussion, the active ratios at 2 × MIC were used to explain the abundance of MA‐ARB.

**Table 1:** Concentrations of antibiotics used in cultivation of faecal microbiota.

Condition	D_2_O (%)	Antibiotic concentration (μg/ml)			Personal medicine history
		Concentration in food^a^	MIC	2 × MIC	
Ctrl	–	–	–	–	
D_2_O	40	–	–	–	
Amox	40	0.080	32	64	V1: 2–3 times a year in childhood; V2: 1–3 times a year in childhood
Ceph	40	0.016	16	32	V1: 1–3 times a year in childhood; V2: 1–2 times a year in childhood
Flur	40	0.09	3	6	a
Tet	40	0.085	16	32	p
Van	40	0	1.2	2.4	×

Note: a: only for veterinary; p: only taken by volunteers’ parents. Amox: amoxicillin; Ceph: cephalexin; Flur: florfenicol; Tet: tetracycline; Van: vancomycin. V1: volunteer 1; V2: volunteer 2.

a (Rettedal *et al*., 2014; Ben *et al*., 2019).

Amoxicillin and cephalexin belong to β‐lactam antibacterial agents, which can inhibit cell wall synthesis and transpeptidation reaction during peptidoglycan formation (Cho *et al*., 2014). The abundances of Amox^R^ in two volunteers were 58% and 40%, respectively, and the abundances of Ceph^R^ of two volunteers were 53% and 30%, respectively. The resistant bacteria ratios of amoxicillin and cephalexin are the highest among the five antibiotics in this study. This result is expected because the ARB to β‐lactam is common in general (Rettedal *et al*., 2014). Especially, amoxicillin and cephalexin are the two most commonly used antibiotics for people living in China (Zhang *et al*., 2015b) and amoxicillin is also the most commonly used antibiotic in livestock industry. The total usage of amoxicillin, including human and animals, is the highest in China (Zhang *et al*., 2015b). Although the volunteers did not take any antibiotics in the past year, amoxicillin and cephalexin were frequently taken as medicine by both donors during their childhood.

Tetracycline is the fourth most used antibiotics in China and a commonly detectable pollutant in wastewater treatment plants (Qiao *et al*., 2018). It has been broadly used for treating urinary tract infection in the past (Chopra and Roberts, 2001). The abundances of Tet^R^ were 25% and 7% in the two volunteers, respectively, lower than those of Amox^R^ and Ceph^R^ (Fig. 2A and B). Although the use of tetracycline has been stopped for human use in China since 1980s, it is still broadly distributed in food and environment (Frazzon *et al*., 2010), which might enrich tetracycline ARB in the gut microbiota. Interestingly, neither of the volunteers has ever taken tetracycline in their lives, although tetracycline was a commonly used medicine in both of their parents’ childhood (Table 1). The volunteers’ parents were born in 1960s and took tetracycline to treat bacterial infections around 1–2 times a year during their childhoods. The grandmother of volunteer 1 still took tetracycline during pregnancy. ARB can vertically transfer from mother to child (Francino, 2015), which is a route to transmit ARG (Becattini *et al*., 2016).

Florfenicol is a particular antibiotic used by veterinarians, and it is the most commonly used veterinary antibiotic in China besides amoxicillin. The disinfection mechanism of florfenicol is affecting protein synthesis by inhibiting of aa‐tRNA binding (Chellat *et al*., 2016). As shown in Fig. 2A and B, florfenicol exposure significantly decreased the proportion of active bacteria in the faecal sample. The abundances of Flor^R^ ARB were 6% and 5.3% of the two volunteers, respectively. Although the volunteers did not use florfenicol‐related medicine directly, Flor^R^ ARB were still present in their gut microbiota to some extent, which may be naturally resistant bacteria or be enriched from food florfenicol exposure.

Vancomycin, as one of the last resort antibiotics, is only used for emergency when other antibiotics cannot provide effective treatment in clinical settings. The concentration of vancomycin in food is almost nil (Table 1), and neither of volunteers nor their parents has a medical history involving the use of vancomycin. This may be an explanation to our results, which no Van^R^ ARB were found in the two volunteers’ gut microbiota (Fig. 2A and B).

The concentration of florfenicol in food is the highest among the five antibiotics used in this study (Table 1). However, the ratio of antibiotic‐resistant bacteria is not directly related to the concentration of the antibiotic in food. Because the concentration of florfenicol in food is as high as 0.09 μg/ml while the concentration of cephalexin in food is much lower (0.016 μg/ml; Ben *et al*., 2019). In contrast, the abundance of Ceph^R^ is the highest. Amoxicillin and cephalexin were often taken to against pathogens during adulthood while florfenicol is not for human. This result suggests that the proportion of ARB exhibited less direct relationship with the concentrations of the antibiotics in food, but is more relevant to the individual exposure history to antibiotics.

Interestingly, there is no difference in the ratios of active bacteria between the low antibiotic treatment (concentrations of antibiotics in food) and the control (Fig. 2A and B). Although the exposure to antibiotics probably has a long‐lasting influence on the gut microbiota (Jernberg *et al*., 2010; Dethlefsen and Relman, 2011), low concentrations of antibiotics that below MIC (Table 1) did not show a significant impact in the selected period of exposure (24 h).

### Comparison of Raman–DIP with traditional cultivation

**Figure 3:**
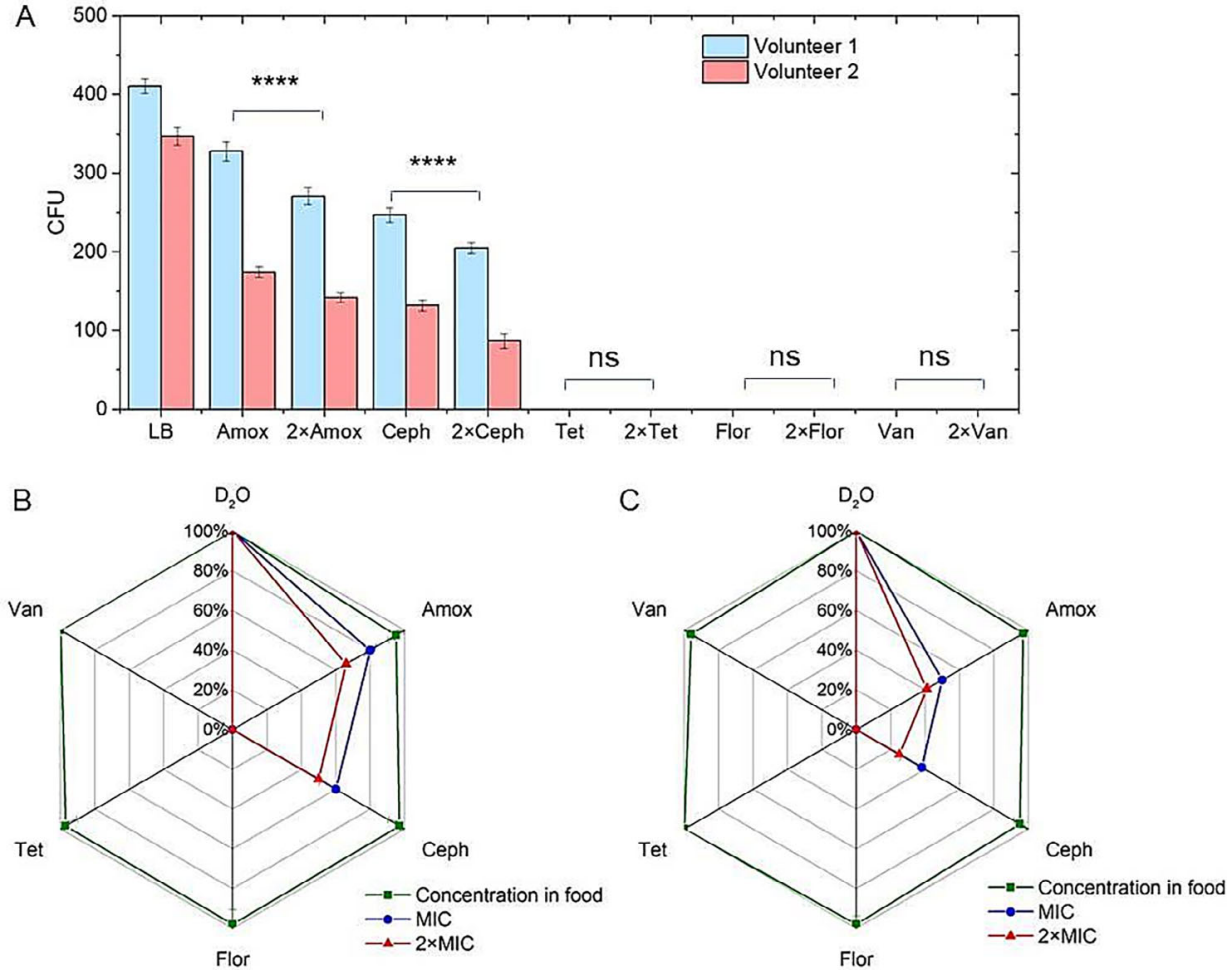
A. CFU counts of intestinal bacteria cultured in LB and LB with different antibiotics at MIC and 2 × MIC.B and C. Percentages of intestinal active bacteria cultured in the LB and the abundance of ARB cultured in LB with different antibiotics at MIC and 2 × MIC from two volunteers. For each condition, three replicates were measured. The error bar represents standard deviation of three samples. Student *t*‐test was used to indicate statistical differences between the CFU of MIC and 2 × MIC. ns indicates none significant difference; *P < 0.05, **P < 0.01, ***P < 0.001, ****P < 0.0001.

We also cultivated the faecal samples on LB agar with different antibiotics. The results showed that the CFU counts of LB medium without any antibiotics was the highest in two volunteers (1.6 × 10^7^ CUF/g ww (wet weight) and 1.4 × 10^7^ CFU/g ww), followed by amoxicillin (1.3 × 10^7^ CUF/g ww and 7.0 × 10^6^ CUF/g ww), 2 × MIC amoxicillin (1.1 × 10^7^ CUF/g ww and 5.7 × 10^6^ CUF/g ww), cephalexin (9.9 × 10^6^ CUF/g ww and 5.3 × 10^6^ CUF/g ww) and 2 × MIC cephalexin (8.2 × 10^6^ CUF/g ww and 3.5 × 10^6^ CUF/g ww) (Fig. 3). There were no CFU counts in LB medium with tetracycline, florfenicol and vancomycin, suggesting that the Tet^R^, Flor^R^ and Van^R^ from the gut microbiota of two volunteers were not culturable in LB medium within 48 h. The CFUs in the plates with 2 × MIC concentrations of amoxicillin and cephalexin were lower than in MIC concentration (*P* < 0.0001).

The CFU counts of gut microbiota on LB agar were about 1.6 × 10^7^ and 1.4 × 10^7^ CFU/g ww of volunteer 1 and volunteer 2. The total number of the bacteria in both gut microbiota by microscope count was about 10^9^/g ww. According to the active ratios measured by Raman–DIP (80% and 63%; Fig. 2), only 2.0%–2.2% of the active bacteria were able to grown on LB agar. Setting the CFU counts in LB medium control (no antibiotics) as 100%, the percentages of CFUs with different antibiotics were calculated and shown in Fig. 3B and C for the two volunteers. These results demonstrated that 41%–66% and 25%–50% of the cultivable human intestinal bacteria in LB were Amox^R^ and Ceph^R^, which were most abundant in the gut microbiota, consistent to the results of Raman–DIP. However, compared with *in situ* Raman test, the CFU counts detected fewer ARB in LB agar because only a small amount of human gut bacteria is able to grow in LB medium. The abundances of ARB tested by CFUs differ to the results of Raman–DIP. However, the CFUs in LB medium with low concentrations of antibiotics were comparable to the CFUs in LB medium control (no antibiotics), suggesting that the antibiotic concentration in food had no effect on the bacteria in short period, which is also consistent to the result of Raman–DIP.

**Figure 4:**
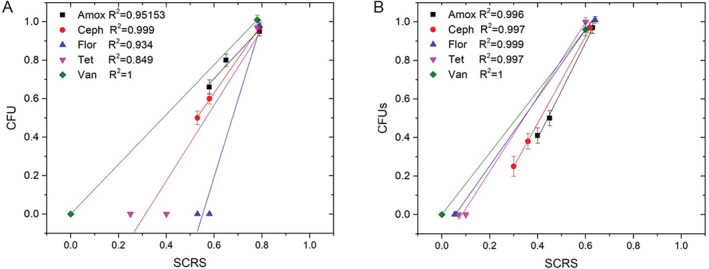
Correlations (A: volunteer 1 and B: volunteer 2) between the ratios of Raman‐active cells and the CFU counts of culturable cells exposed to different antibiotics.

Figure 4 showed the correlation between CFU counts by traditional cultivation and SCRS by Raman–DIP, and the results demonstrated a good agreement between CFUs and SCRS. It shows that many gut bacteria were not readily cultured by the traditional cultivation method (Amann *et al*., 1995; Thursby and Juge, 2017). Therefore, it is difficult to use conventional approach to estimate the actual percentage of ARB in human gut microbiota. It has been previously demonstrated that the addition of D_2_O less than 50% had no significant impact on bacterial metabolism (Berry *et al*., 2015). Therefore, Raman–DIP is able to identify the ARB in the human gut microbiota *in situ*.

### Linking the gap between ARB phenotype and ARG genotype

The locations of individual bacteria on the microscopic slide have been recorded and cells of interest (e.g. ARB) were sorted using RACE (Song *et al*., 2017b). We applied RACE to isolate mini‐metagenomic DNA of Amox^R^, Ceph^R^ and Ceph^S^ MA‐ARB from gut microbiota of both volunteers. The mini‐metagenomic DNA of the 30 sorted single cells were amplified and then sequenced (Supporting Information Fig. S2). According to the DNA sequencing results, the identification of the sorted bacteria is listed in Table 2. Among these sorted bacteria, *Achromobacter* sp., *Delftia* sp., *Flavobacterium* sp., *Massilia* timonae and *Pedobacter* BAL39 were identified as ARB, which have been reported to resist a broad range of antibiotics (Holmes *et al*., 1979; Virginia *et al*., 2006; Preiswerk *et al*., 2011; Sreenivas *et al*., 2014; Hu *et al*., 2015). A strain of *Pseudomonas* sp. was identified as amoxicillin resistant, which has also been reported (Van Craenenbroeck *et al*., 2011). We found that *Acidovorax* sp. and *Staphylococcus* sp. were in the sorted bacteria, which have been reported to resist β‐lactam antibiotics (Miura *et al*., 2013; Rupp and Fey, 2015).

**Table 2:** Identification of bacteria isolated from human faeces by PRECI SCS and subsequent 16S rRNA sequencing.

Sample	Genus	Species	Metabolic active resistance	Pathogen
Amox^R^	*Achromobacter*	Unidentified	Amox	√
	*Acidovorax*	*Acidovorax radicis*	Amox	
	*Delftia*	Unidentified	Amox	√
	*Flavobacterium*	*Flavobacterium sp*. *83* *Flavobacterium soli*	Amox	
	*Massilia*	*Massilia timonae*	Amox	
	*Pedobacter*	*Pedobacter sp*. *BAL39*	Amox	
	*Pseudomonas*	Unidentified	Amox	√
	Unidentified	Unidentified	Amox	
Ceph^R^	*Staphylococcus*	*Staphylococcus capitis* *Staphylococcus epidermidis*	Ceph	√
	Unidentified	Unidentified	Ceph	
Ceph^S^	*Flavobacterium*	Unidentified	/	
	*Pseudomonas*	*Pseudomonas fluorescens*	/	√
	Unidentified	Unidentified	/	

Note: the bacteria marked with red colour are the emerging bacterial pathogens.

According to the pathogen database used in the present study (Li *et al*., 2015), five isolated bacteria were pathogens to human (Table 2), which are *Staphylococcus epidermidis* and *Pseudomonas fluorescens* at the species level, and the unclassified species of *Pseudomonas*, *Delftia* and *Achromobacter* at the genus level. Among them, *Staphylococcus epidermidis*, resistant to cephalexin in the Ceph^R^ sample, was the emerging bacterial pathogen in nosocomial infection (Fulginiti, 1984). As for the treatment of pathogens, Raman‐DIP can help to choose effective antibiotics according to their resistant phenotype and genotype.

**Figure 5:**
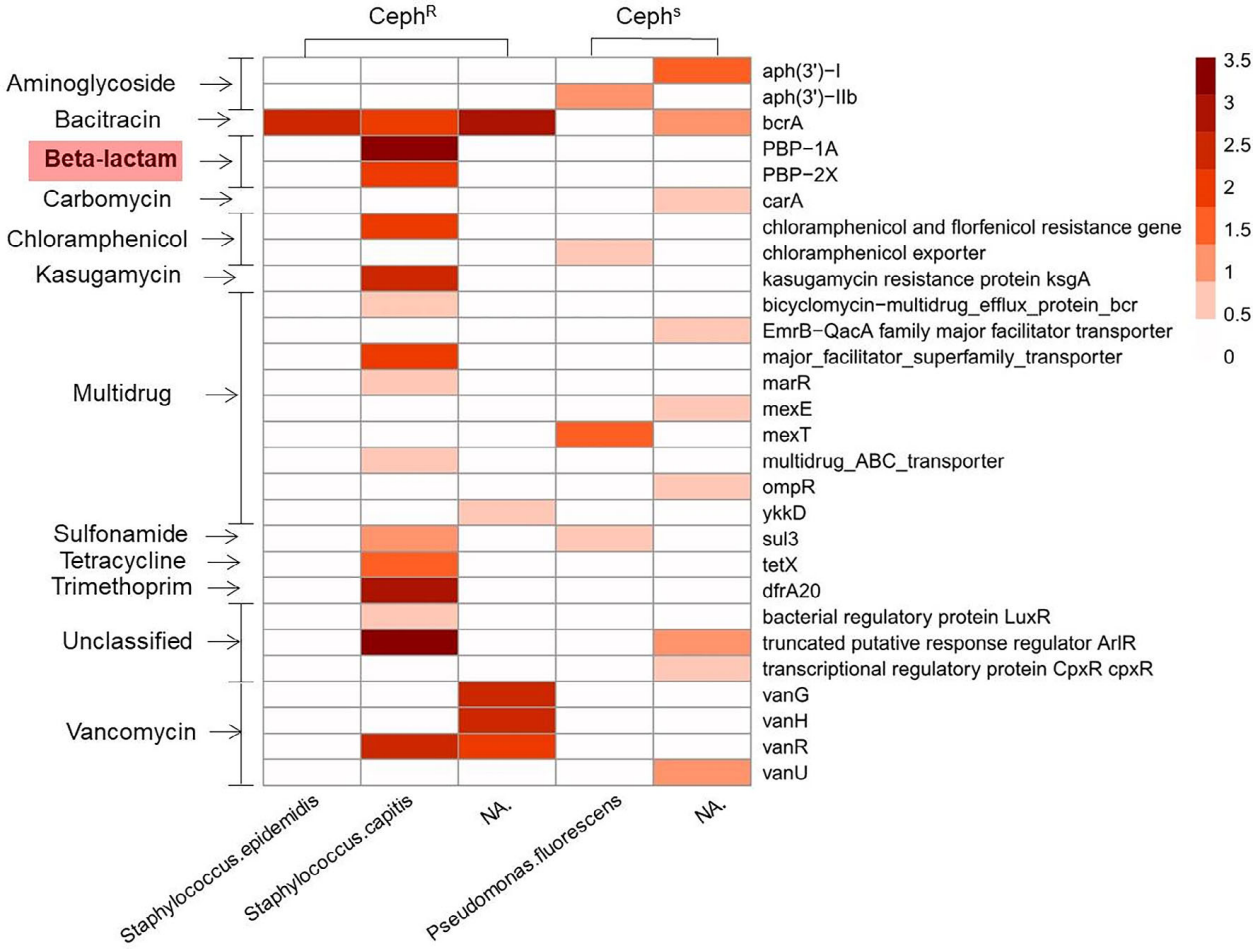
Abundance of ARG subtypes in bacteria from the sorted target MA‐ARB. Beta‐lactam is present in the Ceph^R^ sample but is missing in the Ceph‐sensitive sample.

According to the mini‐metagenomic DNA sequences of 30 sorted cells for Ceph^R^ and Ceph^S^ (Supporting Information Fig. S2), resistant genes in these bacteria were analysed. Figure 5 demonstrated the total 28 ARG subtypes can be categorized into 12 ARG types from two sorted samples. The abundances of individual encoded resistant subtypes ranged from 0 to 3.5, which was calculated using log of the coverage, normalized by the total sequencing data size (copies/Gb). Except for unclassified gene‐encoded subtypes, the CephR sample contain the highest level of ARG genes: vancomycin‐(vanG, vanH, vanR and vanU; subtotal abundance: 6.777), beta‐lactam‐(PBP‐1A and PBP‐2X; subtotal abundance: 5.465) and multidrug‐(bicyclomycin‐multidrug_efflux_protein_bcr, EmrB‐QacA family major facilitator transporter, major_facilitator_superfamily_transporter, marR, mexE, mexT, multidrug_ABC_transporter, ompR and ykkD; subtotal abundance: 4.098). However, no beta‐lactam‐resistant gene was detected in the Ceph^S^ sample. This genotype analysis is in a good agreement with the phenotype analysis by Raman–DIP. The lack of β‐lactam ARG in *Staphylococcus epidermidis* may be due to the fact that the genome of multiple displacement amplification were not fully recovered (Hou *et al*., 2015).

The modification of penicillin‐binding protein 1A (PBP1A) and penicillin‐binding protein 2X (PBP2X) are the most significant PBPs for beta‐lactam resistance (Kosowska *et al*., 2004). In *Staphylococci*, PBP‐1A and PBP‐2X are essential for bacteria growth and survival (Kosowska‐Shick *et al*., 2010). PBP‐1A is the bifunctional enzyme, which is significant to septum formation during the cell division. The amino acid residue 371 and 574–577 of PBP‐1A are responsible to the interaction with beta‐lactam and the altered PBP‐1A is essential for developing high‐level resistance to beta‐lactam (Smith and Klugman, 1998; Contreras‐Martel *et al*., 2006). PBP‐2X and PBP‐1A are able to interact with each other to induce resistance of beta‐lactam (Zerfaß *et al*., 2009). Hence, Ceph resistance from the Raman–DIP analysis (Fig. 1) is consistent to metagenomic data analysis, which showed the presence of ARG (Fig. 5) and associated pathogen (Table 2).

It is worth mentioning that although the abundance of vancomycin‐resistant genes (vanG, vanH, vanR and vanU) was high, we did not detect the presence of active Van^R^ in the Ceph^R^ in the samples (Fig. 5), presumably due to non‐expression of the resistant genes. The expression of genes in cell can be influenced by the external and internal environment such as hormones, metabolism, chemicals, drugs and so on. (Lobo, 2014). Therefore, DNA sequencing is insufficient to reveal behaviour and risk of ARG. It is important to establish a link between phenotypic function and genotype. This technique demonstrated in this study is able to investigate both phenotypes and genotypes of ARG in human gut microbiota.

## Conclusions

In this study, we have applied Raman–DIP, single‐cell sorting and subsequent genomic sequencing to link the phenotypes and genotypes of MA‐ARB in the human gut microbiota at the single‐cell level. Our results demonstrated that there were a large number of MA‐ARBs in the gut microbiota in two healthy individuals. The phenotypes of the MA‐ARB exhibited distinct metabolic activity when exposing to MIC and higher concentrations of antibiotics, detected by the C–D band of SCRS by Raman–DIP. We also found significant differences between the abundances of active bacteria under different concentrations of amoxicillin, cephalexin, tetracycline, florfenicol and vancomycin. We hypothesize that the presence of ARB is related to personal exposure history to antibiotics. We isolated a few ARB bacteria single cells from the gut microbiota according to their SCRS, and performed mini‐metagenomic sequencing. The MA‐ARB and the associated ARG of the two donors were identified. Although ARG of vancomycin‐resistant was found in the isolated MA‐ARB, the bacteria did not show any resistance to vancomycin according to both traditional cultivation and Raman analysis. It suggests that genomic sequencing alone could not be sufficient to reveal the actual ARB in human gut microbiota. Hence, it is important to investigate both genotype and phenotype to uncover the functionality of ARB in gut microbiota. It was the first time that this approach was utilized to study the ARB and ARG in human gut microbiota, paving the way for clinical practice towards precision medicine. This novel method is promising for further study, such as the quality control of human gut microbiota for faecal microbiota transplantation, investigation of drug‐bacteria interaction and the impact of gut microbiota to human diseases.

## Experimental procedures

### Human faeces collection and bacterial growth conditions

All fresh faeces were collected from two healthy adults with the age of 27 and 29 who were volunteers to this study. The volunteers have taken no antibiotics in the past year. The faecal samples were taken immediately and put into sterilized anaerobic tubes within 5 min, avoiding exposure to oxygen in the air. In the anaerobic tubes, the samples were diluted 1:10 with phosphate buffer saline (PBS) solution or supplemented with 40% D_2_O (v/v) and various concentrations of amoxicillin, cephalexin, florfenicol, tetracycline and vancomycin (Table 1, Sigma‐Aldrich). The three antibiotic concentrations represent the typical concentration in food (Ben *et al*., 2019), the minimum inhibitory concentration (MIC) and two times the MIC (2 × MIC), respectively. The MIC was determined according to the averaged MIC of commensal gut bacteria in the Eucast data (http://mic.eucast.org/Eucast2/; Rettedal *et al*., 2014). After cultivating for 24 h at 37°C anaerobically (95% N_2_: 5% H_2_) under shaking (140 rpm), 1 ml sample was collected from each group under different antibiotic concentrations for Raman measurement. Three biological replicates were taken for each sample.

Colony‐counting units (CFUs) were determined in all conditions. Bacteria in faecal sample in PBS were diluted to countable concentrations. Then 50 μl diluted sample was spread onto LB agar plates with amoxicillin, cephalexin, florfenicol, tetracycline and vancomycin with different concentrations (Table 1). These plates were incubated at 37°C anaerobically (95% N_2_: 5% H_2_) for 48 h. The CFUs were counted with triplicates.

### Bacterial purification, single‐cell Raman spectra measurement and analysis

Before Raman measurement, samples were centrifuged at 300 g for 3 min to remove the debris. Then bacteria were harvested by 8000 *g* centrifugation for 5 min to remove supernatants. The cells were collected and washed with deionized water three times to remove the impurities which may interfere with the Raman detection. Cells were then diluted in deionized water to allow observation of single cells under a 100×/0.75 air objective. Afterward, 1.5 μl sample was spotted onto an Aluminium‐coated Raman slide (Xu *et al*., 2017). The total number of the bacteria was counted with five replicates.

SCRS of bacteria from human gut microbiota were acquired by a 532‐nm neodymium‐doped yttrium aluminium garnet (Nd: YAG) laser with a 3.5 mW power and 5 s acquisition time. Spectra were obtained in the range of 400–3200 cm^−1^ with 300 grooves/mm diffraction grating. In each treatment, 80–300 single cells were measured.

All spectral data were preprocessed with Labspec6 software (Horiba) for baseline correction and vector normalization. The ratio of C–D/(C–D + C–H) was calculated by integrating the areas of specific Raman bands i.e. C–D (2040–2300 cm^−1^) and C–H (2800–3100 cm^−1^) in SCRS. This ratio was used as an indicator of the extent of deuterium incorporation by bacteria from D_2_O and their metabolic activities. The relative abundance of antibiotic‐resistant bacteria was determined by counting the SCRS with the presence of C–D bands.

### Raman‐activated single‐cell ejection and genomics

RACS was conducted through single‐cell ejection technique by PRECI SCS (HOOKE Instruments Ltd, China). Based on the principle of laser‐matter interaction, PRECI SCS perfectly achieved label‐free, non‐contact, precise and visualized isolation of single cells from biological samples. Microbial samples (~10^6^ CFU/ml) were loaded onto a sorting chip. RACS has been used to sort six samples, including two amoxicillin‐resistant bacteria (Amox^R^), two cephalexin‐resistant (Ceph^R^) and two sensitive (Ceph^S^) bacteria, according to SCRS with the presence or absence of a C–D band. Each sample contained 20–30 sorted cells. The cells of interest on the chip were isolated from the sorting chip by a laser beam and the sorting process was controlled by PRECI SCS software. The sorted cells were directly ejected into a collector containing cell lysis buffer (Qiagen, Germany). The sorted cells were lysed, and the whole genomic DNA was amplified on‐chip by multiple displacement amplification (MDA) using Phi29 DNA polymerase (Qiagen, Germany). The mini‐metagenomic DNA from the sorted cells was checked by PCR of 16S rRNA using the primer 27F (5′‐AGAGTTTGATCMTGGCTCAG‐3′) and 1492R (5′‐TACGGYTACCTTGTTACGACTT‐3′). According to 16S‐rRNA PCR products, four samples were positive, which were 2 Amox^R^, 1 Ceph^R^ and 1 Ceph^S^ samples. The mini‐metagenomic DNA of these samples were sequenced by the Illumina HiSeq X‐Ten sequencer (Novogene, China).

Six gigabases of sequencing data were generated to each sample. After the quality control, clean reads with an average quality score at least 30 were retained and assembled by using IDBA‐UD default parameter (Lina *et al*., 1999; Peng *et al*., 2012). And the assembled contigs with mincontig 500 bp were reserved. The open reading frames (ORFs) were predicted by Prodigal (v3.2; Hyatt *et al*., 2010). The ARG‐like ORFs were aligned to SARG database (v2.0) using DIAMOND (v1.09; Yin *et al*., 2018) with an e‐value ≤10^−10^. ORFs with at least 70% coverage and 80% similarity were identified as ARG‐like genes. The coverage of ARG‐like ORF in each sample was estimated by mapping clean reads to the contig using BBMap (v36.38) with the cut‐off of 95 similarity and normalized by the data size (copies/Gb; Ma *et al*., 2016). The abundance of each ARG subtype was transformed using logarithmic data size to compare the coverage between different samples. The ARG‐like ORFs on the contigs were sought out the NCBI nr database (released in Mar. 2019) by BLASTP with an E‐value ≤10^−5^. The contigs were annotated as the taxon if more than 50% of the ORFs on the contig were annotated as the same taxon (MEGAN version 5; Mitra *et al*., 2011). The raw data was deposited to NCBI public database with the BioProject number: PRJNA594067.

## Supporting information


**Appendix S1**: Supporting information
