# Supplementary material for: Raman‐activated sorting of antibiotic‐resistant bacteria in human gut microbiota
Source: Environ Microbiol. 2020 Mar 13;22(7):2613–24. doi: 10.1111/1462-2920.14962 (PMC7383503; doi:10.1111/1462-2920.14962)
Supplement: Supplementary file 1 — Appendix S1: Supporting information [file EMI-22-2613-s001.docx]

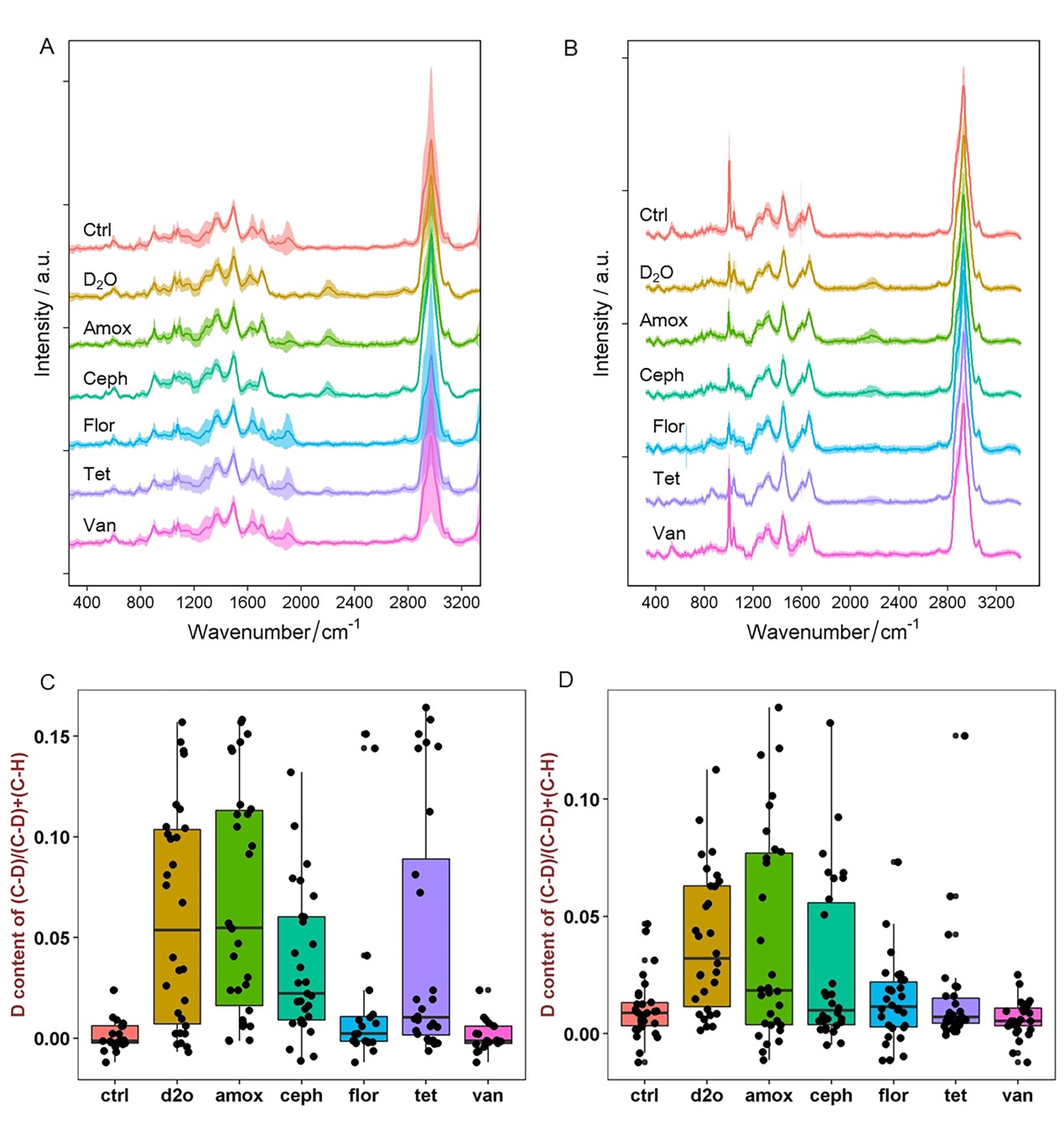


Figure S1 (A) & (B) Averaged SCRS of human intestinal bacteria cultured *in situ* with different antibiotics at 2×MIC from two volunteers. Each spectrum represents an average of SCRS from 80–300 single-cells, and the shadow represents standard deviation. (C) & (D) Boxplots showing single-cell distribution of the intensity ratio of C–D / (C–D + C–H) in SCRS of single cells.


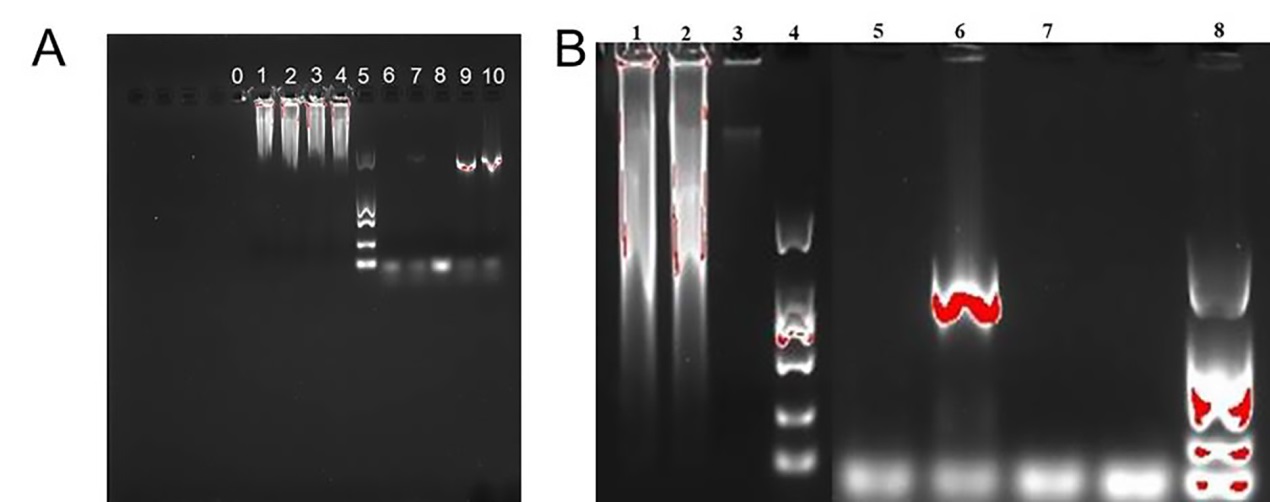


Figure S2 Gel electrophoresis image of the DNA amplification products of the sorted cells. (A) 0 & 6 are negative control without any cells. 5 is the DNA ladder. 1–4 are the electrophoretogram of amplified whole genome of Amox^R^ (1) Ceph^R^ (3) and Ceph^S^ (2&4), while 6-10 are the electrophoretogram of 16S rRNA PCR products of Amox^R^ (7), Ceph^R^ (9) and Ceph^S^ (8&10). (B) 3 & 5 are negative control without any cells. 4 & 8 are the DNA ladder. (1) & (2) are the electrophoretogram of amplified whole genome of Amox^R^ & Ceph^R^, while (6) & (7) are the electrophoretogram of 16S rRNA PCR products of Amox^R^ & Ceph^R^. Each sample contains 30 cells.
